# A Modified mRNA Vaccine Targeting Immunodominant NS Epitopes Protects Against Dengue Virus Infection in HLA Class I Transgenic Mice

**DOI:** 10.3389/fimmu.2019.01424

**Published:** 2019-06-21

**Authors:** Claude Roth, Tineke Cantaert, Chloé Colas, Matthieu Prot, Isabelle Casadémont, Laurine Levillayer, Jessie Thalmensi, Pierre Langlade-Demoyen, Christiane Gerke, Kapil Bahl, Giuseppe Ciaramella, Etienne Simon-Loriere, Anavaj Sakuntabhai

**Affiliations:** ^1^Functional Genetics of Infectious Diseases Unit, Institut Pasteur, Paris, France; ^2^CNRS UMR 2000: Génomique Évolutive, Modélisation et Santé, Institut Pasteur, Paris, France; ^3^Institut Pasteur du Cambodge, Phnom Penh, Cambodia; ^4^Invectys, Paris, France; ^5^Vaccine Programs, Institut Pasteur, Paris, France; ^6^Moderna, Inc., Cambridge, MA, United States; ^7^Beam Therapeutics, Cambridge, MA, United States

**Keywords:** dengue virus (DENV), T cells, vaccine, chimeric vaccine, DNA vaccine, NS epitopes, human HLA transgenic mice

## Abstract

Dengue virus (DENV) induces strong T and B cell responses upon infection. Hence, it is difficult to determine the contribution of cell-mediated immunity alone in the long lasting protection against DENV infection and disease. Numerous CD4+ and CD8+ T cell epitopes have been identified, mainly in the non-structural proteins of DENV. Taking into account the immunogenicity and peptide sequence conservation among the different DENV serotypes, a minimal DENV antigen, called DENV1-NS, has been designed. This antigen is enriched in conserved and highly antigenic epitopes located in the NS3, NS4B, and NS5 regions of DENV1. To evaluate the ability of the DENV1-NS poly-epitope to express the antigenic peptides in the context of different HLA class I molecules, we established its *in vivo* immunogenicity by measuring, after DNA immunization and electroporation, the activation of DENV-specific CD8 T cells in transgenic mice expressing the human HLA-A^*^0201, -A^*^2402, -B^*^0702, and -B^*^3502 class I alleles. We then engineered a lipid nanoparticle (LNP) encapsulated modified mRNA vaccine encoding DENV1-NS and tested immunogenicity and protection in these human HLA class I transgenic mice, after transient blockade of the interferon (IFN) type I receptor. Significant protection was observed, after two injections of the mRNA vaccine. Collectively, these data strongly support the development of T cell-based vaccines targeting immunodominant T cell epitopes that generate potent virus-specific T cell responses conferring immunity against DENV infection.

Dengue virus (DENV) is an arthropod-borne virus transmitted to humans by the mosquito spp., *Aedes aegypti*, and *Aedes albopictus*. Dengue has become one of the most important global public health threats in recent decades, with a global estimate of 3.6 billion people at risk of infection (1). Four DENV (DENV1-4) serotypes co-circulate and are endemic to many tropical and sub-tropical countries, causing 400 million new infections every year, of which 100 million cases are symptomatic, ranging from a self-limiting febrile illness named dengue fever (DF) to more severe life-threatening dengue hemorrhagic fever (DHF) and dengue shock syndrome (DSS). Over 2 million DHF/DSS cases, 500,000 hospitalizations and 25,000 deaths are estimated to occur every year, primarily among children (2). Due to uncontrolled urbanization, increased human migration and the spread of DENV-transmitting mosquitoes, the frequency of epidemics has been constantly growing since the 1960s and the global distribution of dengue has extensively spread.

Dengue fever is characterized by fever with headache, retro-orbital pain, myalgia, arthralgia, rash, abdominal pain, and a viremia that begins 3–4 days following a bite by an infectious mosquito. As the fever subsides at days 4–6 of illness, a fraction of patients develop DHF/DSS characterized by thrombocytopenia, hemorrhagic manifestations, and signs of plasma leakage, which can lead to hypovolemic shock, organ failure and, without appropriate treatment, death (3). Strikingly, while a primary infection with one DENV serotype can induce long-term immunity to homotypic DENV, a subsequent infection by heterotypic serotypes increase the risk of developing severe dengue, a phenomenon postulated to be due to non-neutralizing or sub-neutralizing antibodies. Here, in a process called antibody-dependent enhancement (ADE) serotype-crossreactive immune complexes lead to enhanced infection of FcγR bearing immune cells and/or an increased inflammatory response (4, 5). While type-specific neutralizing responses were initially thought to elicit long-lasting immunity to the primary infecting DENV serotype (6, 7), several cases of symptomatic diseases have been observed following reinfections with the same serotype, which depend on the concentration of pre-existing anti-DENV antibodies (8, 9). Moreover, a strong correlation has been established between the risk to develop severe dengue disease and a specific range of pre-existing anti-DENV antibody titers (10, 11). Thus, both the quality and the quantity of neutralizing anti-DENV antibodies play a crucial role in neutralizing and protecting against dengue infection and disease.

Similarly to the ADE phenomenon, it was proposed, in a scenario termed “original antigenic sin,” that T cells which are primed after a primary DENV infection, can nevertheless undergo clonal expansion upon secondary heterotypic infection, and hinder the specific T cell response against the infecting serotype, making the elimination of DENV-infected cells less efficient (12, 13). These cross-reactive T cells, when stimulated by secondary and heterotypic DENV infection, display an altered cytokine profile, with higher ratios of Tumor Necrosis Factor (TNF) to Interferon gamma (IFN-γ) producing CD4 T cells (14), and high cytokine production but suboptimal degranulation or impaired IFN-γ production (15, 16). However, in spite of these studies, direct evidence for a role of cross-reactive T cells in the pathogenesis of severe dengue disease is lacking, and in fact, recent data strongly support a protective role for serotype-specific and cross-reactive T cells against DENV infection (17). Among the arguments supporting a beneficial role of T cells, a strong correlation was established between the protection against severe dengue, the expression of certain Human Leukocyte Antigen (HLA) alleles and a polyclonal memory CD8^+^ T cell response with a high magnitude in healthy dengue-immune individuals (18–24). Likewise, in a recent study from Cambodian children, a role for T cells in protection from clinical dengue was further supported, with a higher activation of Natural Killer (NK) cells and T cells observed in strictly asymptomatic dengue-infected individuals, compared to clinical dengue patients, whereas the up-regulation of gene expression pathways leading to plasmablast development and the secretion of DENV-specific antibodies correlated with the development of clinical dengue (25).

In humans, both CD4 and CD8 T cells contribute to protection against DENV, with CD4 T cells mainly targeting structural proteins Capsid (C) and Envelope (E) and the non-structural protein NS1, and CD8 T cells preferentially targeting non-structural proteins NS3, NS4B, and NS5 (24, 26–29). Given the expression of sub-neutralizing or cross-reactive antibodies and the enhanced risk of severe dengue following vaccination among seronegative vaccine recipients (30) or after multiple natural infection episodes, a vaccine candidate has been designed on its ability to induce a strong T cell response. This vaccine, called DENV1-NS, is composed of the most immunogenic regions of NS3, NS4B, and NS5. DNA immunization with a plasmid encoding DENV1-NS in mice expressing different human HLA class I molecules confirmed the induction of a strong CD8 T cell response against peptides derived from these NS regions. Using this strategy, and with the intention to develop an effective T cell-based vaccine, we report here that a prime-boost immunization of human HLA class I transgenic mice with low dose of a modified mRNA encoding DENV1-NS induces a strong T cell immunity, with a significant protection against DENV1 infection, after transient blockade of the IFN type I receptor (31–33), in the absence of neutralizing or sub-neutralizing anti-DENV antibodies. These data clearly demonstrate the validity of the approach using minimal NS-derived T cell epitopes in the induction of a protective immunity against dengue infection and disease.

## Materials and Methods

### Mice and Infections

HLA-A^*^0201, -A^*^2402, -B^*^0702, and -B^*^3501 monochain transgenic/H-2 null mice on the C57BL/6 background (34–36) were bred in the Institut Pasteur facility and used between 6 and 10 week of age. All animals were intraperitoneally inoculated with 2 mg of anti-ifnar1 antibody (clone MAR1-5A3, Interchim, France) (33) 24 h prior to DENV inoculation. For all experiments, mice were infected by retro-orbital injection of 10^6^ PFU DENV1 KDH0026A, in 200 μl PBS, then bled on days 1, 2, 3, and 6 after virus inoculation, for measurement of viremia and sacrificed on day 7 post-infection. All mouse experiments were performed following Institutional animal care and use committee-approved animal protocols.

### Design of the Nucleotide Sequence Encoding DENV1-NS Poly-Epitope

**Figure 1:**
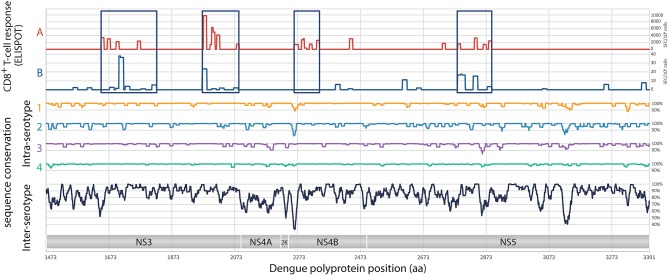
Analysis of target regions for T cells and sequence conservation of non-structural proteins of DENV. Analyses of the magnitude of T cell responses, in SFC/10^6^ cells **(A)**, from Weiskopf et al. (24), and in SFC/10^6^ cells **(B)**, from Rivino et al. (28). Intra-serotypes and inter-serotypes sequence conservation are represented for DENV1 (yellow line), DENV2 (blue line), DENV3 (purple line), DENV4 (green line), and for all DENV serotypes (black lines), respectively.

Given the identification of CD8+ T cell epitopes from previously infected donors (24, 28, 37), 4 regions have been selected in the non-structural proteins NS3, NS4B, and NS5, for a total of 540 amino acids: 2 regions in NS3 (185 and 134 amino acids, respectively), 1 region in NS4B (86 amino acids) and 1 region in NS5 (135 amino acids) (Figure 1). Based on the sampled genetic diversity of the 4 serotypes of DENV, and with the idea that T cell epitopes are either conserved among different serotypes, or are serotype-specific with nevertheless the ability to induce cross-reactive CD8+ T cell responses, a prototype consensus sequence based on epidemic strains of DENV1 has been selected, the DENV1-NS T cell poly-epitope (patent WO2015/197565, initially filled on June 23, 2014) (Figure 1). A total of 2,033 full-length DENV genome sequences (865 for serotype 1, 1,663 for serotype 2, 427 for serotype 3 and 63 for serotype 4) were aligned using MAFFT (38), with manual adjustments according to the amino acid sequence. Mean pairwise sequence identity was evaluated at each position intra- and inter-serotypes at the nucleic and the amino-acid levels. Analysis of the concatenated regions of interest revealed strong intra-serotype conservation, and generally a higher degree of sequence identity compared to the genome as a whole, including for inter-subtypes comparisons. A serotype 1 consensus sequence was selected, as it presented the highest average sequence identity with the 4 serotypes (vs. serotype 1: 99.48%; serotype 2: 83.48%; serotype 3: 89.39%; serotype 4: 76.95%). For DNA immunization, the optimized nucleotide sequence encoding DENV1-NS poly-epitope was cloned in the pcDNA3.1 plasmid, under the CMV promoter. For mRNA vaccination, a lipid nanoparticle (LNP) encapsulated modified mRNA vaccine encoding DENV1-NS was prepared as described previously (39).

### Generation of Modified mRNA and LNP

Chemically modified mRNA was synthesized *in vitro* by T7 RNA polymerase-mediated transcription from a linearized DNA template containing an open reading frame flanked by a 5′ and 3′UTR and a polyA tail. Uridine was fully replaced with N1-methylpseudouridine, and Cap1 was utilized to increase mRNA translation efficiency.

Lipid nanoparticle formulations were generated as described previously (40). Briefly, lipid stocks were dissolved in ethanol at a molar ratio of 50:10:38.5:1.5 (ionizable lipid: helper lipid: structural lipid: PEG-lipid) and mixed with mRNA at a ratio of 3:1 (mRNA:lipid). mRNA-loaded nanoparticles were exchanged into final storage buffer and had final particle sizes of 80–100 nm, >80% encapsulation, and <10 EU/mL endotoxin.

### Immunizations

Before immunization, mice were first anesthetized with a mix solution of xylazine 2% (Rompun, Bayer santé, Loos, France) and ketamine 8% (Imalgen 1000, Merial, Lyon, France) in PBS through the intraperitoneal (I.P.) route according to individual animal weight and duration of anesthesia. DNA immunization was performed following a prime boost administration regimen at 3-week interval by intradermal (I.D.) injection of plasmid DNA pcDNA3.1 encoding the DENV1-NS sequence (each injection consists of 2 simultaneous I.D. injections of 50 μg plasmid DNA in the lower back followed by *in vivo* electroporation) and spleen cells were harvested 10 days after the boost. The electroporation settings, using the AgilePulse apparatus (BTX, Harvard apparatus, MA, USA) consist of 3 Voltages groups: 450 V, with a pulse length of 50 microseconds, a pulse interval of 0.2 microseconds and 1 pulse; 450 V, with a pulse length of 50 microseconds, a pulse interval of 50 milliseconds and 1 pulse; and 110 V, with a pulse length of 10 milliseconds, a pulse interval of 20 milliseconds and 8 pulses. The immunization with mRNA was performed following a prime boost regimen at 3-week interval or 4-week interval, by intramuscular (I.M.) injection of LNP-based mRNA encoding the DENV1-NS sequence or irrelevant mRNA as a negative control (each injection consists of one I.M. injection of 10 or 2 μg mRNA vaccine encoding the DENV1-NS sequence or 10 μg irrelevant mRNA). For quantification of the T cell responses following mRNA immunization, the prime boost regimen was performed at 3-week interval, and spleen cells were harvested 7 days after the boost. For the evaluation of the vaccine efficacy, the prime boost regimen was performed at 4-week interval, and mice were challenged with the virus 4 weeks after the boost.

### Viruses and Cell Lines

The *in vivo* and the *in vitro* assays were conducted using the DENV1 KDH0026A strain (provided by Dr. L. Lambrechts, Institut Pasteur, Paris). The DENV1 virus was grown using the Aedes Albopictus mosquito cells line C6/36 cultured in Leibovitz's L-15 medium supplemented with 10% fetal bovine serum containing 0.1 mM non-essential amino acids and 1x tryptose phosphate broth. Vero-E6 cells were provided by Dr. M. Flamand (Institut Pasteur, Paris).

### Quantification of Viral Loads

For assessment of viremia, blood was collected by retro-mandibular puncture in EDTA coated-microvette tubes (Fisher Scientific, Illkirch, France). After centrifugation, plasma samples from DENV-infected mice were extracted with the RNeasy Mini Kit (Qiagen). DENV RNA levels were determined by TaqMan one-step quantitative reverse transcriptase PCR (qRT-PCR) on a QuantStudio 12K Flex Real-time PCR system (Life Technologies) using standard cycling conditions. Viral burden is expressed on a log_10_ scale as viral RNA equivalents per milliliter after comparison with a standard curve produced using serial 10-fold dilutions of DENV1 RNA from known quantities of infectious virus. The following primer sets were used:

Forward: 5′-GGAAGGAGAAGGACTCCACA-3′;

Reverse: 5′-ATCCTTGTATCCCATCCGGCT-3′;

Probe: 5′-(FAM) CTCAGAGACATATCAAAGATTCCAGGG-3′ (MGB).

### Synthetic Peptides

All peptides were synthesized by Proimmune Ltd (Oxford, UK), as crude material, and were tested individually in Elispot assay and in intracellular cytokine staining for IFN-γ and TNF-α secretion. The peptides used in this study and corresponding to DENV1 sequences or to their DENV2, 3 or 4 variants are listed in Table 1.

**Table 1:** DENV Peptides and serotypes variants used in this study.

**Peptide^a^**	**Sequence^b^**	**Serotype**	**NS region^c^**	**HLA restriction^d^**	**References^e^**
**p30**	**RYLPAIVREAI**	DENV1, 2, 3	NS3.1	A*3101/B*0702/A*0201	(28) / (41)
p30 (DV4)	RILPISIVREAL	DENV4		B*0702/B*3501	IEDB
**p36**	**APTRVVASEM**	DENV1	NS3.1	B*0702	(41)
P36 (DV2, 3, 4)	APTRVVAAEM	DENV2, 3, 4		B*0702/B*3501	(28, 41) / (24)
**p49**	**TPEGIIPAL**	DENV1, 3	NS3.2	B*3501/B*0702	(24, 36) / (24)
p49 (DV2)	**TPEGIIPSM**	DENV2		B*3501/B*0702	(24)
p49 (DV4)	**TPEGIIPTL**	DENV4		B*3501/B*0702	(24)
**p50**	**LPVWLSYKVA**	DENV1, 4	NS3.2	B*5301/B*5101	(24)
p50 (DV2)	**LPVWLAYRVA**	DENV2		B*5101	(24)
p50 (DV3)	**LPVWLAHKVA**	DENV3		B*3501	(24)
**p32**	**QYSDRRWCF**	DENV1	NS3.2	A*2402	IEDB
p32 (DV2.1)	**NYADRRWCF**	DENV2		A*2402	(28)
p32 (DV2.2)	NYADRKWCF	DENV2		A*2402	IEDB
p32 (DV3)	KYTDRKWCF	DENV3		A*2402	IEDB
p32 (DV4)	SYKDREWCF	DENV4		A*2402	IEDB
**p17**	**LDARTYSDPLALREFKEF**	DENV1	NS3.2	B*3501	IEDB
p17 (DV2)	LDARIYSDPLALKEFKEF	DENV2		A*2402	(28, 29)
p17 (DV3)	LDARTYSDPLALKEFKEF	DENV3		B*3501	IEDB
p17 (DV4)	LDARVYADPMALKDFKEF	DENV4		B*3501	(24)
**p21**	**HPASAWTLY**	DENV1, 3	NS4B	B*3501	IEDB
p21 (DV2, 4)	RPASAWTLY	DENV2, 4		B*0702/B*3501	(41)
**p51**	**TLYAVATTI**	DENV1, 4	NS4B	A*0201	IEDB
p51 (DV2)	**TLYAVATTF**	DENV2		A*2402	IEDB
p51 (DV3)	**TLYAVATTV**	DENV3		A*0201	(42)
**p33**	**ITPMMRHTI**	DENV1	NS4B	A*2402	IEDB
p33 (DV2)	VTPMLRHSI	DENV2		B*0702	(41)
p33 (DV3)	ITPMLRHTI	DENV3		A*2402	IEDB
p33 (DV4)	LTPMLRHTI	DENV4		–	–
**p56**	**SMVNGVVKL**	DENV1, 4	NS5	A*0201	IEDB
p56 (DV2)	SMGNGVVRL	DENV2		A*0201	(42)
p56 (DV3)	SMINGVVKLL	DENV3		A*0201	IEDB
**p15**	**KPRICTREEF**	DENV1	NS5	B*0702	(24)
p15 (DV2)	TPRMCTREEF	DENV2		B*0702/B*3501	(24, 41) / (24)
p15 (DV3)	KPRLCTREEF	DENV3		B*0702	(24)
p15 (DV4)	NPRLCTREEF	DENV4		B*0702	(24)

a *peptides in bold are derived from the Poly DENV1-NS sequence used for immunization, and tested by Elispot*.

b *sequences in red are peptides variants corresponding to DENV2, 3, or 4 sequences, and sequences in bold are peptides tested in this study*.

c *The NS3.1, NS3.2, NS4B, and NS5 regions are derived from DENV serotype 1, serotype 2, serotype 3 and serotype 4 (Genbank accession number NP_059433.1, NP_056776.2, YP_001621843.1 and NP_073286.1, respectively)*.

d *The HLA restriction was determined from responding human donors or human HLA transgenic mice, or predicted from the Immune Epitope Database and Analysis Resource (www.iedb.org), with a percentile rank <0.2. HLA restrictions are shown in bold when they were also identified in this study*.

e *Except a few peptides predicted from the Immune Epitope Database and Analysis Resource (www.iedb.org), all peptides used in this study were previously identified in humans or in HLA class I transgenic mice, using ELISpot assay*.

### IFN-γ ELISPOT Assay

Mouse spleen cells were collected and depleted of red blood cells and IFN-γ-producing splenocytes were quantified by ELISPOT assays after a 24 h period of stimulation with peptides, as already described (43). Briefly, 96-well nitrocellulose-backed plates (Multiscreen, Merck-Millipore, Molsheim, France) were coated with anti-mouse IFN-γ mAb at 5 μg/ml (BD Pharmingen, France) in 50 μl of 50 mM carbonate buffer (pH 9.6) overnight at 4°C. Wells were blocked with 200 μl of complete medium at room temperature for 2 h and then washed three times with serum-free medium. The coated wells were filled in quadruplicate with splenocytes from immunized mice in complete α-MEM medium with individual peptides at 2 μg/ml and incubated at 37°C for 20 h. After incubation, the wells were washed three times with PBS-0.05% Tween 20, and then incubated with 50 μl biotinylated anti-mouse IFN-γ (BD bioscience, France) at 1 μg/ml in PBS Tween for 1 h at room temperature. The spots were developed using streptavidin-alkaline phosphatase (Mabtech, Stockholm, Sweden) and BCIP/NTB substrate (Promega, Madison, MI, USA) and counted using an automated ELISPOT reader (Immunospot, Cellular Technology Limited, Cleveland, OH, USA). The number of IFN-γ-producing cells was expressed as spot-forming cells (SFC) relative to 1 × 10^6^ spleen cells. Values were calculated by subtracting the number of spots detected in non-stimulated control wells. Values were considered positive if they were equal to or >20 spots and at least three times above the means of the unstimulated control wells. As positive wells, cells were stimulated with ConA at 10 μg/ml (Sigma Aldrich, Lyon, France).

### Intracellular Cytokine Staining

Spleen cells from immunized mice (100 μl/well in 96 U-bottom plates) were incubated with individual peptides (adding 50 μl at 4 μg/ml for 2 h, and 50 μl Golgi Stop at 4 μl/ml for 4 h), in complete medium containing 10% FCS. Cells were then harvested, membrane stained using anti-mouse CD4-V500 (BD Biosciences), CD3-alexa488 (BD Biosciences), and CD8-PerCP (eBioscience) antibodies, and intracellular staining was performed using the intracellular staining kit (BD Biosciences) and anti-mouse IFN-γ-APC and TNF-α-eFluor 450 antibodies (eBioscience).

### Virus Neutralization Assay

The quantification of neutralizing activity of antibodies against DENV was determined using a flow cytometry-based assay, as described previously (44). Briefly, 2-fold serial dilutions of plasma samples were incubated at 37°C for 1 h with a 1/200 dilution of virus inducing 15–20% infection. Virus -antibody mixture was then added to 5 × 10^4^ Vero cells for 2 h at 37°C, after which cells were washed with fresh medium and incubated for 24 h at 37°C. Cells were then fixed with 4% paraformaldehyde, stained using the intracellular staining kit (BD Biosciences) with 4G2 antibody conjugated to Alexa Fluor^TM^ 488, and the percentage of infected cells was measured by flow cytometry. Plasma samples from mice immunized with mRNA CT vaccine and from *ifna*r^−/−^ mice injected intravenously with 10^6^ pfu DENV1 were used as negative and positive controls, respectively.

### Statistical Analyses

Differences between 2 groups were evaluated using non-parametric Mann-Whitney U-test in the assessment of the immunogenicity and the immune protection or unpaired *t*-test in the evaluation of the expansion and the contraction phases of the T cell response. All computer analyses were performed using GraphPad Prism 7 (GraphPad Software Inc. La Jolla, CA, USA). *P* < 0.05 were considered statistically significant.

## Results

### Immunogenicity of the DENV1-NS Poly-Epitope

Given the identification of CD8^+^ T cell epitopes from human donors and to minimize the dilution of the immune response observed following DENV infection, a minimal DENV antigen has been designed from conserved and highly antigenic T cell epitopes. Using compiled DENV T cell epitope distribution and strength (24, 28, 37, 41) and the consensus prediction method available through the IEDB Analysis Resource (available at: www.iedb.org), we have selected four regions from a DENV serotype 1 consensus that are enriched in CD8 epitopes: two regions in NS3, one in NS4B, and one in NS5 (Figure 1). The resulting DENV1-NS poly-epitope corresponds to the concatenation of amino acid positions 1650-1829, 1959-2092, 2262-2347, and 2766-2899 based on the reference sequence NP_059433. Within serotypes, pairwise identity along the genome ranged between 0.878 and 1 for DENV1, 0.816 and 1 for DENV2, 0.847 and 1 for DENV3 and 0.94 and 1 for DENV4.

**Figure 2:**
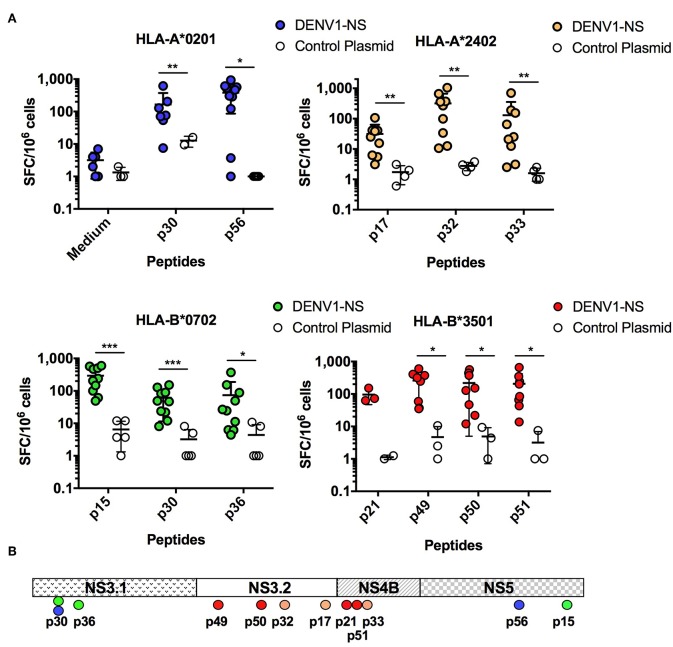
Quantification of the T cell responses in HLA-A*0201, -A*2402, -B*0702, and -B*3501 transgenic mice by ELISpot assay. **(A)** Three independent experiments were performed, in which a total of 10 and 4 HLA-A*0201 transgenic mice received the DENV1-NS construct and the control plasmid (CT), respectively, 9 and 4 HLA-A*2402 transgenic mice received the DENV1-NS construct and the control plasmid, respectively, 10 and 5 HLA-B*0702 transgenic mice received the DENV1-NS construct and the control plasmid, respectively, and 8 and 3 HLA-B*3501 transgenic mice received the DENV1-NS construct and the control plasmid, respectively. All the animals were immunized by intradermic injection (100 μg DENV1-NS or control plasmid) followed by *in vivo* electroporation. Two immunizations were performed at 3-week interval, and spleen cells were tested for IFN-γ secretion by ELISpot 10 days after the second injection. Individual mice were tested in parallel with different peptides at 2 μg/ml and with concanavalin A (ConA) at 5 μg/ml, final concentration. Lines represent mean and SEM. Differences between mice immunized with the DENV1-NS construct and the control plasmid were evaluated using non-parametric Mann-Whitney U-test (*p < 0.05, **p < 0.01, ***p < 0.001). **(B)** Schematic representation of the T cell epitopes from the DENV1-NS poly-epitope, which induce a significant T cell response in HLA transgenic mice. NS3.1, NS3.2, NS4B, and NS5 represent the 4 antigenic regions selected in the DENV1-NS poly-epitope.

To determine whether the DENV1-NS poly-epitope can be produced endogenously and then processed and presented in the context of human HLA class I molecules, we performed DNA-based immunization combined with electroporation (EP) in HLA-A^*^0201, -A^*^2402, -B^*^0702, and B^*^3501 monochain transgenic/H-2 null mice (34, 36), which are associated with low T cell responses (for the HLA-A^*^0201 and -A^*^2402 alleles) and high T cell responses (for the HLA-B^*^0702 and -B^*^3501 alleles) (24), and represent the most frequent alleles in Caucasoid, Oriental and Amerindian ethnic groups, from the allele frequency Net Database website (available at: *www.allelefrequencies.net*). The animals were immunized by intradermal (I.D.) injection of plasmid DNA expressing the DENV1-NS under a CMV promoter and the interferon (IFN)-γ response of spleen cells was quantified by Enzyme Linked Immunospot (ELISpot) assay against individual peptides. Among the potential epitopes expressed by DENV1-NS, and predicted from the Immune Epitope Database and Analysis Resource (www.iedb.org) to bind to HLA-A^*^0201, or shown in literature to induce a significant T cell response in the context of HLA-A^*^0201 molecules (41, 42, 45), 2 antigenic peptides were identified, which elicit a strong T cell response in the HLA-A^*^0201 transgenic mice: p30 and p56 located in the NS3.1 and the NS5 regions of the DENV1-NS, respectively (Figure 2 and Table 1). In the immunized HLA-A^*^2402 transgenic mice, 3 peptides were also identified: p17 and p32 located in the NS3.2 region and p33 located in the NS4B region (Figure 2). These results confirm previous identification of HLA-A^*^0201- or HLA-A^*^2402-restricted epitopes, as targets for T cell responses in humans (16, 28, 29, 37, 46), or predicted to bind strongly these alleles (www.iedb.org). Finally, 3 and 4 peptides have been identified in HLA-B^*^0702 and HLA-B^*^3501 transgenic mice, respectively, with a higher number of antigenic peptides inducing a stronger T cell response in the HLA-B^*^3501 mice, in comparison with HLA-B^*^0702 transgenic mice (Figure 2). These data clearly show that the DENV1-NS poly-epitope is expressed and processed correctly, resulting in the presentation of antigenic peptides, which elicit a strong T cell response in the context of different HLA molecules. They also show that all the antigenic peptides identified in DENV1-NS correspond to T cell epitopes previously identified from human donors or from HLA transgenic mice infected with DENV.

### An mRNA Vaccine Encoding DENV1-NS Poly-Epitope Induces Strong CD8 T Cell Activation

**Figure 3:**
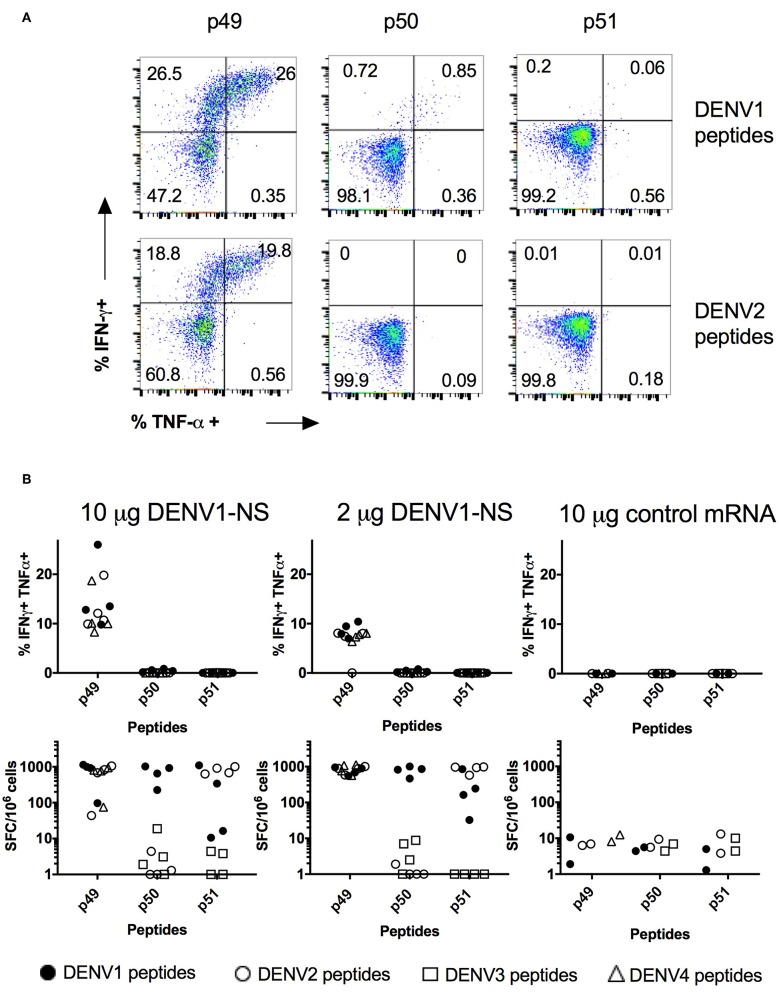
Quantification of the T cell responses in HLA-B*3501 transgenic mice by intracellular cytokine staining and ELISpot assay. Two groups of 4 HLA-B*3501 transgenic mice received an intramuscular inoculation of 10 or 2 μg of the LNP-based mRNA encoding DENV1-NS and 1 group of 2 HLA-B*3501 transgenic received 10 μg of the LNP-based irrelevant mRNA as a negative control. Two immunizations were performed at 3-week interval and spleen cells were tested 8 days after the second immunization for IFN-γ and TNF-α secretion by intracellular staining, after 6 h *in vitro* stimulation with peptides, in the presence of Golgi stop or by ELISpot assay after 20 h stimulation with peptides. **(A)** Flow cytometry analyses of gated CD3+, CD8+, for IFN-γ and TNF-α secretion. Plots show CD8 T cell responses from one representative animal against p49, p50, and p51 derived from DENV1 sequence (upper panels) or from their DENV2 variants (lower panels) after immunization with 10 μg of the LNP-based mRNA encoding DENV1-NS. **(B)** Comparison of the T cell responses analyzed by intracellular cytokine staining (upper panels) and by ELISpot assays (lower panels). Closed circles: peptides derived from DENV1. Open circles: peptide variant derived from DENV2. Open squares: peptide variant from DENV3. Open triangles: peptide variant from DENV4.

Intramuscular delivery of LNP with encapsulated modified mRNA revealed strong immunogenicity *in vivo* (39). Therefore, we inoculated HLA transgenic mice with the LNP-based mRNA vaccine encoding DENV1-NS. Eight to 10-week old HLA-B^*^3501 transgenic mice were divided into 3 groups, which received an intramuscular inoculation of 10 or 2 μg of the mRNA encoding DENV1-NS or 10 μg of an irrelevant mRNA as a negative control. After a booster immunization at day 28, spleen cells from immunized animals were harvested 8 days later, at day 36, and the frequency of CD8 T cells secreting IFN-γ and TNF-α and spleen cells producing IFN-γ was evaluated by intracellular staining and by ELISpot assay, respectively. Both assays were used in parallel, as they provide complementary information, in particular by allowing the identification of responding cells with intracellular cytokine measurement by flow cytometry, and the detection of small number of IFN-γ-producing cells in response to poorly immunogenic peptides with the ELISpot assay which is more sensitive. Flow cytometry analyses of responding CD8 T cells after 6 h *in vitro* stimulation with the HLA-B^*^3501-restricted peptides p49, p50, or p51 revealed different capacity of these peptides to stimulate CD8 T cells, with 26% of CD8 T cells producing IFN-γ and TNF-α- against p49, whereas only 0.85 and 0.06% of CD8 T cells produce both cytokines in response to p50 and p51, respectively (Figure 3A). As the amino acid sequence of the antigenic peptides may vary between the 4 DENV serotypes, with a few amino acid substitutions located in the HLA binding sites (anchor residues) (Table 1), we asked whether mice immunized with the DENV1-NS vaccine could recognize peptide variants, which are derived from heterotypic DENV serotypes. As shown in Figures 3A,B, both the p49 peptide (TPEGIIPAL for DENV1, 3) and its 2 serotype variants (TPEGIIPSM and TPEGIIPTL for DENV2 and DENV4, respectively) stimulate CD8 T cells to produce IFN-γ and TNF-α. We then compared the frequency of T cells responding to the DENV1-specific p50 and p51 peptides, with the frequency of cells responding to the serotype variants of these peptides, using the ELISpot assay, which is more sensitive than flow cytometry. Results show that while p50 and p51 induce a significant T cell response, none of the 2 DENV serotype variants of p50 (LPVWLAYRVA for DENV2 or LPVWLAHKVA for DENV3) stimulate T cells, and only 1 serotype variant of p51 (TLYAVATTF for DENV2) can stimulate T cells. Interestingly, the DENV2 variant of p51 is more potent in stimulating T cells than the DENV1-specific p51 peptide, both for the 10 μg and the 2 μg dose of DENV1-NS vaccine (Figure 3B). Altogether, these data show that immunization with mRNA vaccine encoding DENV1-NS induces a strong stimulation of CD8 T cells, which in some cases, could be highly cross-reactive against heterotypic peptides.

### Efficient Maturation of CD8+ T Cells After Late Transient Blockade of Type I IFN Signaling

Most mouse models for dengue infection and disease make use of mice lacking type I or type I and type II IFN receptors, rendering them highly susceptible to DENV infection (47). However, most of these models are not relevant to study the role of memory and effector memory T cells in the immune protection against DENV infection. For instance, type I IFN plays a major role in controlling the CD8 T cell response to viral infection, notably by blocking the expansion of pre-existing non-specific memory T cells (out-of-sequence signaling), while promoting the proliferation of antigen-specific CD8 T cells at the beginning of the response (in-sequence signaling) (48). Importantly, this regulation depends essentially on the timing of type I IFN exposure relative to T cell receptor signaling. In this context, we developed a mouse model, which allows the clonal expansion of antigen-specific T cells, while retaining DENV susceptibility. This was achieved by injecting, 1 day before the challenge with DENV, the anti-IFNAR antibody to block transiently the type I IFN signaling, as already reported for WNV and ZIKV infection in wild type mice (32, 49). The benefit of this approach is that it ensures the normal development of memory T cell responses, because blockade of type I IFN signaling is performed just before the challenge, once the immune response is established.

**Figure 4:**
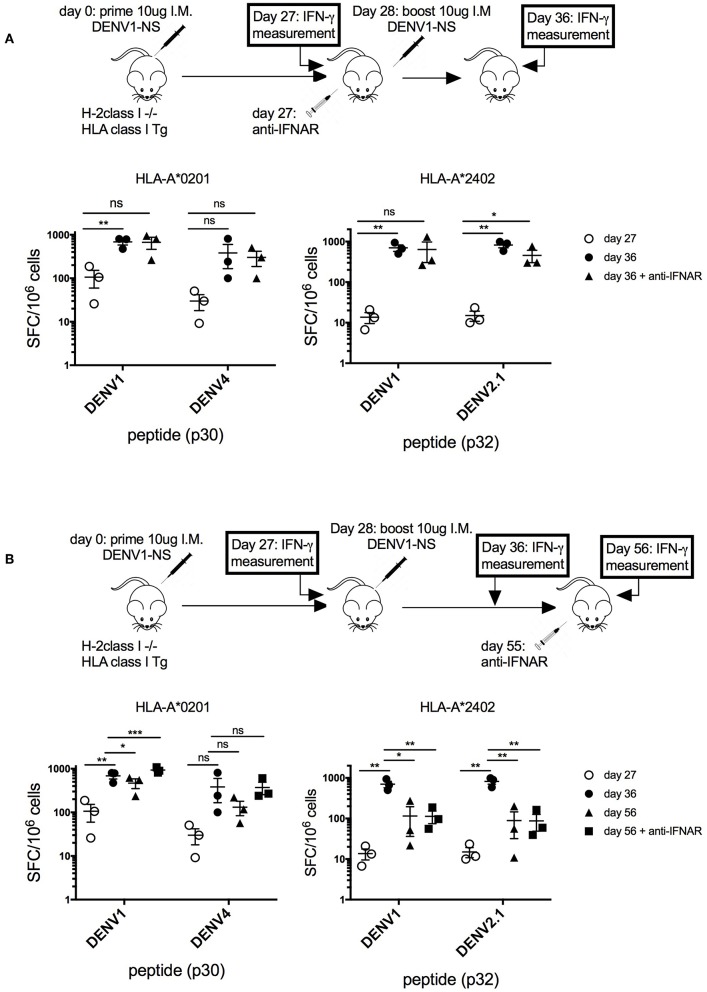
Effect of transient blockade of type I IFN signals on the expansion and contraction phases of antigen-specific T cells. **(A)** Three groups of 3 HLA-A*02:01 transgenic mice and 3 groups of 3 HLA-A*24:02 transgenic mice received 2 intramuscular inoculations of 10 μg of the LNP-based mRNA encoding DENV1-NS at 4-week interval. One group of mice received in addition 1 intraperitoneal inoculation of 2 mg anti-IFNAR antibody at day 27, just before the boost. Spleen cells were tested by ELISpot assay for IFN-γ secretion at days 27 and 36, after *in vitro* stimulation with the p30 peptide from DENV1 or its peptide variant from DENV4 for HLA-A*0201 transgenic mice or the p32 peptide from DENV1 or its peptide variant from DENV2.1 for HLA-A*2402 transgenic mice. Mean and SEM are shown. Differences in the ELISpot responses between 2 groups were evaluated using unpaired *t*-test (ns, non-significant, *p < 0.05, **p < 0.01). **(B)** Three groups of 3 HLA-A*0201 transgenic mice and 3 groups of 3 HLA-A*2402 transgenic mice received 2 intramuscular inoculation of 10 μg of the LNP-based mRNA encoding DENV1-NS at 3-week interval. One group received in addition 1 intraperitoneal inoculation of 2 mg anti-IFNAR antibody at day 55. Spleen cells were tested by ELISpot assay for IFN-γ secretion at days 27, 36, or 56. Mean and SEM are shown. Differences in the ELISpot responses between 2 groups were evaluated using unpaired *t*-test (ns, non-significant, *p < 0.05, **p < 0.01, ***p < 0.001).

Thus, to verify that the transient blockade of type I IFN signaling, performed at a late stage after the prime with the antigen (at day 27 after the immunization), does not prevent the establishment of a memory T cell response, we quantified T cell responses before and after the boost, with and without blocking IFN response (Figure 4A). Quantification of the T cell response in HLA-A^*^0201 and HLA-A^*^2402 transgenic mice revealed a 7-fold and a 50-fold increase in the frequency of p30- and p32-specific T cells, respectively, between days 27 and 36, confirming the expansion of antigen-specific T cells after the boost at day 28. The same T cell proliferation was observed in response to p30 (DENV4) or p32 (DENV2) peptides corresponding to serotype variants (Figure 4A). More importantly, the same proliferation was observed in these transgenic mice, with or without anti-IFNAR treatment, showing that depletion of type I IFN signaling at a late stage does not prevent the proliferation of antigen-specific T cells, which expand after the boost (Figure 4A). To assess also the effect of anti-IFNAR treatment on the contraction phase of effector T cells, the antibody was administered at a late stage after the boost (at day 55), and peptide-specific T cell responses were measured in HLA-A^*^0201 and -A^*^2402 treated or not with the antibody.

As shown in Figure 4B, in HLA-A^*^0201 transgenic mice, the anti-IFNAR treatment does not prevent but rather increases the proliferation of p30-specific T cells, whereas it does not modify the proliferation of p32 or p32 (DENV2.1)-specific T cells in HLA-A^*^2402 mice at day 56, after the contraction phase. This result shows that, while a transient blockade of type I IFN signals in HLA-A^*^0201 can increase the proliferation of peptide-specific T cells, it does not prevent the contraction phase of peptide-specific T cells in HLA-A^*^2402 mice, which is required for the development of memory T cell responses (Figure 4B).

### Vaccine Efficacy in Human HLA Transgenic Mice

Given the potential to induce strong T cell responses against several NS epitopes, we wanted to assess efficacy of the mRNA vaccine encoding DENV1-NS, in immunocompetent mice expressing different HLA class I molecules. The ability to elicit immunity and protection against DENV infection was tested in the HLA-A^*^0201, -A^*^2402, and B^*^3501 transgenic mice, which developed a strong T cell response against different peptides located in the NS3, NS4B and NS5 proteins. The animals were vaccinated following a prime-boost immunization, with a boost at day 28 after the prime, followed by a treatment with anti-IFNAR antibody at day 55 and a challenge at day 56 with the DENV1 strain KDH0026A, derived from a clinical isolate.

**Figure 5:**
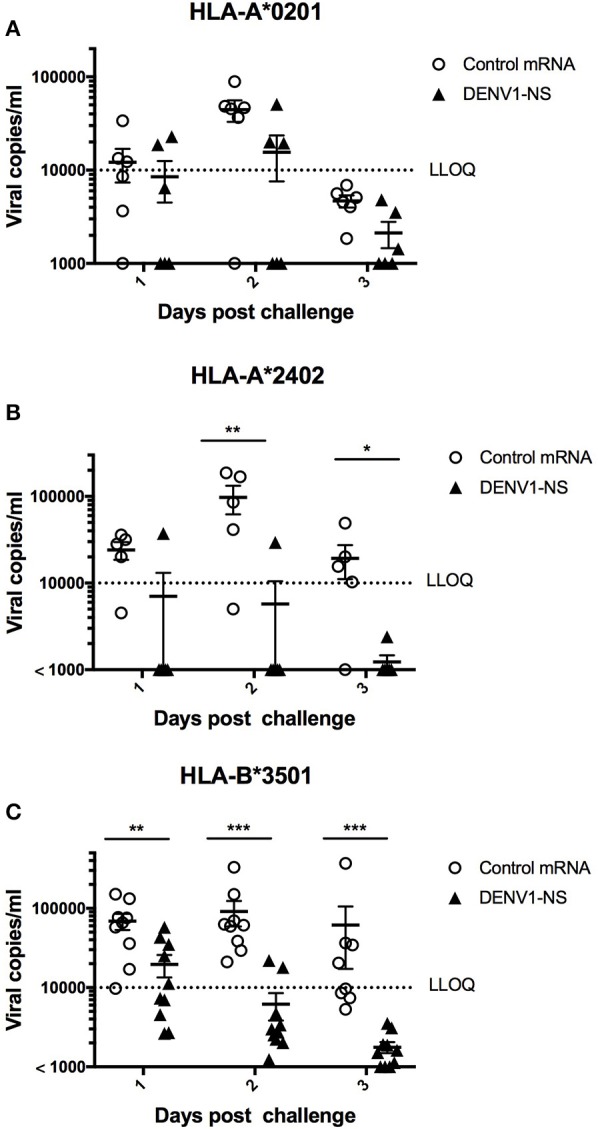
Immune protection induced by immunization with the LNP-based mRNA encoding DENV1-NS. **(A)** Two groups of 6 HLA-A*0201 transgenic mice were immunized with the LNP-based mRNA encoding DENV1-NS or control mRNA, **(B)** Five and six HLA-A*2402 transgenic mice received the LNP-based mRNA encoding DENV1-NS and control mRNA, respectively, **(C)** Nine and ten HLA-B*3501 transgenic mice received the LNP-based mRNA encoding DENV1-NS and control mRNA, respectively. Mice were immunized by intramuscular injection of 10 μg LNP-based mRNA encoding DENV1-NS or control mRNA, at day 0 and at day 28. One day prior challenge with the virus, mice received 1 intraperitoneal inoculation of 2 mg anti-IFNAR antibody (MAR1-5A3) and they were challenged at day 56 by retro-orbital injection of 10^6^ pfu DENV1 (KDH0026A strain). Quantification of virus replication in plasma samples was performed by qRT-PCR at days 1, 2, and 3 after the challenge. LLOQ: Lower limit of quantitative detection. Lines represent mean and SEM. Differences between mice immunized with the DENV1-NS vaccine and the CT vaccine were evaluated using non-parametric Mann-Whitney U-test (*p < 0.05, **p < 0.01, ***p < 0.001).

In the HLA-A^*^0201 mice, 5 out of 6 mice immunized with the control mRNA vaccine developed a significant viremia, at day 2 after the challenge, whereas 3 out of 6 mice vaccinated with the mRNA encoding DENV1-NS revealed a detectable viremia, of which 2 mice had viremia values close to the lower limit of quantitative detection (LLOQ) (Figure 5A). More strikingly, 5 out of 6 vaccinated HLA-A^*^2402 mice revealed no viremia at days 1, 2, and 3 after the challenge, whereas 4 out of 5 HLA-A^*^2402 mice immunized with the control mRNA developed a significant viremia until day 3 (Figure 5B). Finally, 8 out of 10 vaccinated HLA-B^*^3501 mice revealed no detectable viremia at day 2 after the challenge, whereas 9 out of 9 HLA-B^*^3501 mice immunized with the control mRNA vaccine developed a significant viremia at day 2 (Figure 5C). At day 3, no viremia was detected in vaccinated HLA-B^*^3501 animals, whereas 5 out of 8 HLA-B^*^3501 mice immunized with the control mRNA vaccine still had significant viremia, indicating that even in case an initial viremia is developed in vaccinated mice, it is resolved faster than in non-vaccinated animals (Figure 5C). Finally, it should be also noted that none of the vaccinated or control mice died after DENV infection.

To rule out the possibility that the immune protection induced by DENV1-NS vaccination could be mediated in part by neutralizing antibodies, plasma samples from vaccinated mice were tested for their neutralizing potential against DENV infection *in vitro.* Using a flow cytometry-based assay, as described previously (50), we thus analyzed the ability of immune serum from mice vaccinated with DENV1-NS to neutralize DENV1 infection of Vero cells. While serum from immunocompromised mice lacking type I IFN signaling (*ifan*r^−/−^ mice) and infected with DENV1 can efficiently neutralize DENV1 infection, plasma samples from HLA-B^*^3501 mice immunized either with mRNA DENV1-NS vaccine or with CT mRNA did not reveal any neutralizing activity against DENV1 infection, even at a high concentration (Supplementary Table 1). These results confirm the absence of neutralizing antibodies induced after DENV1-NS vaccination, which could mediate protection against DENV1 infection. Taken together, these data demonstrate the efficacy of a T-cell based vaccine, which targets the immunodominant T cell epitopes from NS proteins, in the induction of a protective immunity against DENV infection and disease.

## Discussion

In this study, we have shown that vaccination of HLA transgenic mice with a LNP-encapsulated mRNA encoding a minimal antigen, enriched in conserved and highly antigenic epitopes from NS3, NS4B, and NS5 regions of DENV1 induces a potent T cell response and a protection against DENV1 infection. Lipid nanoparticles (LNP) have been be already used for the delivery of small interfering RNA (siRNA) and they are currently being evaluated in late-stage clinical trials via intravenous administration (51). Moreover, the LNP-based, modified-mRNA vaccine platform generates robust and protective immune responses in different animal models, including mice and monkeys (52). In the light of these results, we took advantage of this technology to elicit a potent T cell response against several DENV peptides and, to assess CD8 T cell-induced protection in the context of different HLA class I backgrounds.

With the limitation that CD8 T cells were only identified by intracellular staining, the strong T cell responses detected by ELISpot, supports a role for these CD8 T cells in the immune protection against DENV infection. This is also in agreement with previous reports showing the induction of peptide-specific responses in HLA transgenic mice that provided protection against DENV challenge (53). Four different HLA class I transgenic strains were selected for measuring the T cell response against DENV peptides, and for the evaluation of the immune protection against a challenge with DENV: the HLA-A^*^0201, -A^*^2402, and HLA-B^*^0702 and -B^*^3501 which represent the most frequent alleles in Caucasoid, Oriental and Amerindian ethnic groups, and are associated with low and high response frequency and magnitude, respectively (24). Depending on the antigenic peptides recognized by T cells, and the positions of amino acid substitutions expressed in the different DENV serotypes, serotype-specific T cells or cross-reactive T cells were stimulated, which target peptides from both homotypic as well as heterotypic DENV strains. Contrary to previous observations showing that cross-reactive T cells could display an altered cytokine profile upon stimulation with peptide from serotypes variants, T cells from the HLA-B^*^3501 transgenic mice immunized with the mRNA vaccine did not reveal any difference in the cytokine profile following *in vitro* stimulation with the DENV2 and 4 serotype variants of p49. Further analyses of the cytokine profile and the ratio of TNF to IFN-γ producing CD8 T cells in response to more peptides and their serotype variants, at different concentrations, should confirm the unaltered T cell response to peptides from serotypes variants, in comparison with peptides derived from DENV1. Taking into account this limitation, quantification by ELISpot of the T cell response to the p30 and p32 peptides or to their serotype variants revealed the same percentage of IFN-γ-producing cells, strongly suggesting the activation of cross-reactive T cells that recognize different DENV serotypes. Further analyses of the T cell responses against other heterologous peptides, such as p56 DENV2, 3 in HLA-A^*^0201, p17 DENV2, 3 and 4 in HLA-A^*^2402 and p15 DENV2, 3 and 4 in HLA-B^*^0702 transgenic mice, should confirm the activation of cross-reactive T cells induced following DENV1-NS immunization, which target conserved epitopes, in accordance with the high genetic identity between the 4 DENV serotypes in the selected DENV1-NS sequence. Importantly, such cross-reactive T cells were also identified in DENV-immune donors infected with ZIKV, after *in vitro* stimulation with the APTRVVAAEM peptide from DENV2, 3, 4, or ZIKV or the APTRVVASEM peptide from DENV1, which corresponds to the p36 peptide derived from the DENV1-NS poly-epitope, and restricted by the HLA-B^*^0702 molecule (54). Further experiments in HLA class I transgenic mice are required to determine whether vaccination with the DENV1-NS poly-epitope induces significant protection against other DENV serotypes in the context of different HLA class I molecules.

In a recent study from naturally infected human donors it was shown that the pattern of the immunodominant CD8 T cell epitopes differs according to the DENV serotype, with DENV3-specific responses predominantly targeting structural proteins, whereas DENV1-, DENV2- and DENV4-specific responses are mainly directed against non-structural proteins (55). In this context, it would be important to determine whether the DENV1-NS poly-epitope, which contains only immunodominant epitopes from non-structural proteins, can nevertheless induce the activation of effector CD8+ T cells which can mediate immune protection against the other DENV serotypes, including more specifically DENV3.

Since immunization with the DENV1-NS mRNA vaccine induces protective immunity at least 1 month after the boost, phenotypic analyses of tetramer-positive CD8+ T cells in different HLA transgenic mice should confirm the induction of memory T cells after DENV1-NS vaccination. A more precise identification of the different peptides from DENV1 or from the other DENV serotypes and a comparison in their capacity to induce and to maintain such memory T cells would be helpful to refine the sequence of the DENV1-NS poly-epitope and thus to improve the immune protection in the context of multiple DENV infections and in different HLA backgrounds. In this sense, a more detailed map of the distribution and strength of the CD4 and CD8 T cell epitopes from DENV1-NS, by quantifying the IFN-γ responses with overlapping peptides, should confirm a hierarchy in the strength of the T cell responses in different HLA backgrounds, which should coincide with the immune protection, as observed in humans and transgenic mice expressing the protective HLA-DRB1^*^0401 and the HLA-B^*^0702 or -B^*^3501 class II and class I alleles, respectively (24, 56).

In addition to inducing strong T cell responses to non-structural proteins, with an immune memory, the advantage of our strategy resides in the absence of neutralizing antibody induction against E and/or prM-protein, hence the absence of possible vaccine induced antibody-dependent enhancement. More specifically, the aim of this study was not to compare the efficiency of a vaccine targeting T cells vs. a vaccine targeting B cells and inducing neutralizing antibodies, either alone or in combination with this T cell vaccine on protection against infection, but rather to show that the activation of T cells, in the absence of neutralizing antibodies is efficient in inducing an immune protection against DENV infection. These results are consistent with previous studies showing a protective role for T cell-mediated immunity against DENV infection, in the presence or in the absence of anti-DENV neutralizing antibodies (53, 57). Similar protection was also observed in the absence of neutralizing anti-DENV antibodies after immunization of IFN-α/βR^−/−^ mice with antigenic peptides from DENV or BALB/c mice or African green monkeys with recombinant capsid protein (58, 59). Although we did not observe any enhanced DENV infection in vaccinated animals, infection experiments *in vitro* with FcγR bearing target cells in the presence of different concentrations of immune serum should allow us to confirm the absence of enhancing antibodies induced after vaccination with the DENV1-NS poly-epitope.

In summary, our results highlight the fact that a minimal poly-epitope, containing immunodominant T cell epitopes from NS3, NS4B, and NS5 non-structural proteins of DENV1 is able to induce a potent CD8 T cell response against DENV1 peptides, resulting in a protective immunity against DENV1 infection. Future studies will be directed to confirm the immunogenicity and protective efficacy of the DENV1-NS or other DENV T cell poly-epitope vaccines against infection with different DENV serotypes in transgenic mice expressing different HLA class I and class II molecules and in other animal models.

## Ethics Statement

This study was carried out in accordance with the recommendations of the Ethical committee: CETEA under number 2014-0001, and the CHSCT committee under number 12.454 at the Institut Pasteur, Paris, France.

## Author Contributions

CR and ES-L designed the DENV1-NS poly-epitope. CR and KB designed the research. CR, CC, MP, IC, and LL performed the experiments. ES-L and AS provided valuable scientific discussion in the field of flavivirus virology. PL-D, JT, KB, CG, TC, GC, and AS provided valuable scientific discussion and expertise in the field of vaccinology. CR wrote the paper.

### Conflict of Interest Statement

CR, ES-L, and AS are inventors on a patent filing (patent WO2015/197565, initially filled on June 23, 2014) related to this work. KB and GC are current or previous employees of Moderna, Inc. and receive salary and stock options as compensation for their employment. GC is employed by Beam Therapeutics, and JT and PL-D are current or previous employees of Invectys. The remaining authors declare that the research was conducted in the absence of any commercial or financial relationships that could be construed as a potential conflict of interest.
