# Supplementary material for: A Modified mRNA Vaccine Targeting Immunodominant NS Epitopes Protects Against Dengue Virus Infection in HLA Class I Transgenic Mice
Source: Front Immunol. 2019 Jun 21;10:1424. doi: 10.3389/fimmu.2019.01424 (PMC6598640; doi:10.3389/fimmu.2019.01424)
Supplement: Supplementary file 1 [file Table_1.pdf]

Supplementary Table 1 | Neutralization activity (Neut50) of plasma samples against DENV1[a]

Exp.	Immunization	Mouse #1	Mouse #2	Mouse #3	Mouse#4
1[b]	Control mRNA	<100	<100	<100	<100
	DENV1-NS	<100	<100	nd	nd
2[c]	Control mRNA	<100	<100	<100	<100
	DENV1-NS	<100	<100	<100	<100
3[d]	DENV1	940	1000	920	nd

[a]: The values in each cell correspond to the dilution of plasma sample giving 50% inhibition of DENV1 infection on Vero cells. [b]: Two groups of 4 and 2 HLA-B*3501 transgenic mice received a prime boost regimen with 10µg control mRNA and DENV1-NS vaccine, respectively, at 4-week interval and plasma samples were tested individually for neutralization activity against DENV1 infection 4 weeks after the boost. [c]: Two groups of 4 HLA-B*3501 transgenic mice received a prime boost regimen with 10µg control mRNA and DENV1-NS vaccine, respectively, at 4-week interval and plasma samples were tested for neutralization against DENV1 infection on Vero cells 4 weeks after the boost. [d]: Plasma samples from *ifnar*-/- mice obtained 4 weeks after intravenous inoculation with 10^6 pfu DENV1 KDH0026A. nd: not done.
